# Anaesthesia and physiological monitoring during *in vivo* imaging of laboratory rodents: considerations on experimental outcomes and animal welfare

**DOI:** 10.1186/2191-219X-2-44

**Published:** 2012-08-09

**Authors:** Jordi L Tremoleda, Angela Kerton, Willy Gsell

**Affiliations:** 1 Biological Imaging Centre (BIC), Medical Research Council (MRC) Clinical Science Centre, Imperial College London, Hammersmith Campus, Cyclotron Building, Du Cane Road, London, W12 0NN, UK; 2 Central Biological Services, Imperial College London, South Kensington, London, SW7 2AZ, UK

**Keywords:** Preclinical imaging, Anaesthesia, Physiological monitoring

## Abstract

The implementation of imaging technologies has dramatically increased the efficiency of preclinical studies, enabling a powerful, non-invasive and clinically translatable way for monitoring disease progression in real time and testing new therapies. The ability to image live animals is one of the most important advantages of these technologies. However, this also represents an important challenge as, in contrast to human studies, imaging of animals generally requires anaesthesia to restrain the animals and their gross motion. Anaesthetic agents have a profound effect on the physiology of the animal and may thereby confound the image data acquired. It is therefore necessary to select the appropriate anaesthetic regime and to implement suitable systems for monitoring anaesthetised animals during image acquisition. In addition, repeated anaesthesia required for longitudinal studies, the exposure of ionising radiations and the use of contrast agents and/or imaging biomarkers may also have consequences on the physiology of the animal and its response to anaesthesia, which need to be considered while monitoring the animals during imaging studies. We will review the anaesthesia protocols and monitoring systems commonly used during imaging of laboratory rodents. A variety of imaging modalities are used for imaging rodents, including magnetic resonance imaging, computed tomography, positron emission tomography, single photon emission computed tomography, high frequency ultrasound and optical imaging techniques such as bioluminescence and fluorescence imaging. While all these modalities are implemented for non-invasive *in vivo* imaging, there are certain differences in terms of animal handling and preparation, how the monitoring systems are implemented and, importantly, how the imaging procedures themselves can affect mammalian physiology. The most important and critical adverse effects of anaesthetic agents are depression of respiration, cardiovascular system disruption and thermoregulation. When anaesthetising rodents, one must carefully consider if these adverse effects occur at the therapeutic dose required for anaesthesia, if they are likely to affect the image acquisitions and, importantly, if they compromise the well-being of the animals. We will review how these challenges can be successfully addressed through an appropriate understanding of anaesthetic protocols and the implementation of adequate physiological monitoring systems.

## Review

### Introduction

The use of imaging technologies is increasing in biomedical research due to their great scope for non-invasively studying biochemical and biological processes in the living animal. Their application represents a major impact on the refinement of *in vivo* studies in animal models, in particular for allowing longitudinal monitoring of the onset and the progression of disease within the same animal, and studying the biological effects of drug candidates and their therapeutic effectiveness. They provide a very useful set of tools for a more rapid, efficacious and cost-effective use and characterisation of animal disease models, with great potential for translational research. Indeed, small animal imaging is extensively used as a preclinical experimental tool in many animal models of human diseases including cardiovascular [1], neurodegenerative [2] and musculoskeletal disorders [3] and cancer studies [4]. More recently, it has been applied for the development of stem cell-based therapies [5,6]. There have also been advances wherein researchers are increasingly using imaging for phenotyping and characterisation of transgenic disease models [7].

The main challenge for *in vivo* imaging remains the ‘biological motion’ not only regarding the physical restrain, but also the respiratory and cardiac activities affecting the quality of the images. In contrast to human studies, imaging of small animals generally requires anaesthesia which helps to restrain the animals and their gross motion, but there is still the need to control cardiac and respiratory motion. During anaesthesia, there is an inevitable autonomic nervous system depression which induces cardiovascular and respiratory depression and induces hypothermia. In addition, other conditions such as repeated anaesthesia required for longitudinal studies, the exposure of ionising radiations and the use of contrast agents and/or imaging biomarkers will also have consequences on the physiology of the animal and therefore will need considerations. All these factors will have a profound effect on the animal’s homeostasis and may thereby confound the image quality and interpretations. Therefore, it is important to implement adequate non-invasive monitoring systems appropriately suited for the different imaging modalities.

Anaesthesia plays a key role in animal imaging, and thus, investigators who are planning imaging experiments are required to be familiarised with the multitude of anaesthesia protocols commonly used for imaging and the physiological monitoring systems available. Various imaging modalities are used for small animal imaging, including magnetic resonance imaging (MRI), computed tomography (CT), positron emission tomography (PET), single photon emission computed tomography (SPECT), high frequency ultrasound and optical imaging techniques such as bioluminescence and fluorescence imaging. While all these modalities are implemented for *in vivo* imaging, there are certain differences in terms of animal handling and preparation for imaging, how the monitoring systems are implemented and, importantly, how the imaging procedures themselves can affect mammalian physiology.

We will review the anaesthesia protocols commonly used in laboratory animals during *in vivo* imaging, providing a brief description of the most commonly used imaging modalities, discussing different anaesthesia protocols and general procedures associated with the preparation of the animal (e.g. transport of animals to imaging facility, acclimatisation period, fasting and diet, administration of any contrast agent) as well as reviewing the challenges associated with the implementation of these physiological monitoring systems during preclinical imaging and how this can be addressed to minimise the impact on the well-being of the animals and its effects on image acquisition and outcome of the studies.

### Imaging modalities: considerations for animal handling

A number of imaging modalities are available for preclinical research, all of them providing a common advantage of allowing longitudinal non-invasive serial imaging studies within the same animals and ability to investigate any anatomical-structural and/or functional alterations within the tissue/organs of the animal. However, it is important to understand how the implementation of these techniques may influence the animal’s physiology and its handling during imaging. We will initially revise the particularities of each modality on the impact of animal handling and its physiological monitoring (for detailed comparison on preclinical imaging technologies, see reviews from Koba et al. [8] and Kagadis et al. [9]).

CT is an X-ray-based imaging modality in which an X-ray source and a detector mounted on a gantry rotate around the specimen to be imaged. The animal is exposed to multiple X-rays through different angles to produce three-dimensional (3D) images of the anatomical structures [10]. Currently available preclinical CT scanners can achieve high resolution with an isotropic voxel size of as low as a few micrometres (down to 5 to 10 μm), providing good contrast images for mineralised tissues (bone), but it is poor for imaging other non-mineralised tissues. However, the use of contrast agents that are based on heavy elements such as iodine or barium allows good imaging enhancement of the different soft-tissue anatomical compartments [11].

CT technology remains extensively used in orthopaedic research and also for characterising anatomical phenotypes in transgenic animals. It is also used for co-registering anatomical imaging with functional data from PET/SPECT modalities and for attenuation correction for these radionuclide-based techniques.

One of the main challenges of this technology is to achieve a reasonable resolution and sensitivity in small field of view within a short acquisition time, accounting for time that animal is required to be under anaesthesia, and also to minimise radiation exposure. Most of the acquisitions should not require high radiation; however, levels could become critical during high-resolution imaging with higher energy X-rays, longer acquisition times and higher frequency imaging. Small animal CT causes radiation doses ranging from 70 to 400 mGy [12]. Doses between 6.5 to 7 Gy are lethal to mice, but even lower doses have shown biological effects (e.g. stimulation DNA repair, free-radical detoxification). Therefore, researchers should be aware of the risk of interference due to radiation during imaging.

The speed of acquisition is also important when contrast agents are used since most of these agents are rapidly cleared away from the bloodstream by the kidneys as rodents typically have a high heart rate (heart rate of mice ranges between 400 and 600 bpm; in rats, between 250 to 400 bpm compared with 60 to 80 bpm for adult humans) and a very short circulation time (approximately 10 s in mice compared with approximately 30 s in humans). Animal imaging technology is already implemented with gating-based acquisition systems, in which image acquisition is acquired simultaneously and triggered at some predefined physiological signals or processed post-acquisition. This allows controlling for the interference effects due to the physiological movement created through the cardiac and respiratory cycle and has remarkably improved the quality of the images acquired. Ongoing challenges are focusing on the development of ultrafast acquisitions and further improvements on detector sensitivity. The benefits of applying gating during image acquisitions have been well reported in micro-CT imaging of rodents [13].

MRI is a non-ionising 3D imaging technique that has advantages over other methods that use ionising radiation such as CT, SPECT and PET, especially for serial imaging. This technology uses strong magnetic field to align the spins of hydrogen nuclei within the tissues and then the application of pulses of radio waves to systematically alter this alignment, causing the hydrogen nuclei to produce a rotating magnetic field that is detectable by the scanner. This signal is then manipulated by additional magnetic fields to be digitalized and to build up enough information to construct an image of the targeted area of the body [14]. Nowadays, preclinical systems can routinely achieve a resolution of 100 μm in all dimensions in living animals, providing high-quality anatomical imaging with good structural detail. Moreover, this technology can also provide information on chemical composition (spectroscopy) and other physiological functional parameters such as blood flow velocity [15]. These techniques are extensively used for anatomical, functional and physiological characterisations of tissues/organs in preclinical models.

The ongoing challenges for preclinical MRI are also related to the relative small anatomical size of rodents compare to humans; for a mouse image to retain the same anatomical definition as that achieve in human images, the acquisition must be with a voxel volume (3D imaging pixel unit volume) approximately 3,000 times smaller than that of a human and somehow compensating for the signal deficit. The two most important ways to overcome these problems are by optimising the radio-frequency coil that surrounds the sample and elicits and receives the nuclear magnetic signal and by employing longer acquisition times. Another method to increase the signal is to work with higher magnetic fields, and generally, the preclinical systems are working on the range of 7 to 9.4 T, commonly going up to 11.7 T [14].

The handling of animals and their anaesthesia regimes becomes challenging when carrying out functional MRI, in which the response to a stimulus is assessed through alterations in the animal physiology. In such studies, it is crucial to maintain the animal within stable and narrow well-defined physiologic parameters to allow for the critical detection of a physiologic response associated to the stimulus. Anaesthesia can markedly affect such functional imaging procedures by affecting the blood flow, blood oxygenation levels and cardiac and respiratory functions [16]. The need to use longer imaging times for some MRI acquisitions represents a major challenge for these studies, and researchers must ensure that adequate physiological monitoring is supported with gating systems to minimise biological motion effects and that the animal’s homeostasis through the scanning period is maintained. Because of the high magnetic field, monitoring equipment must be specifically built-in without containing any ferromagnetic material. There are few suppliers that provide MRI-compatible equipment including electrocardiogram (ECG), temperature, blood pressure and respiration monitoring equipment [17,18]. All the anaesthesia and monitoring equipment used should not emit radio frequencies that interfere with the scan. MRI-compatible systems using fibre-optic or carbon fibre cabling avoid this problem. Main power supplies can also carry interference through the radio-frequency screen; therefore, monitoring equipment should use an adequately filtered and isolated power source or be battery powered [19].

PET and SPECT rely on the detection of photons emitted from radiolabelled tracers in the body. SPECT systems record gamma rays directly after radionuclide emission through collimated detectors rotating around the animal. Most preclinical SPECT scanners are equipped with multi-pinhole collimators which allow acquisitions with higher spatial resolution and sensitivity. A tomographic data reconstruction is applied, yielding a 3D dataset that can then be manipulated to show any particular axis of the body [20]. PET systems also detect gamma rays. The radionuclides emit positrons which rapidly annihilate with an electron in the tissue while emitting two photons in opposite directions. The two simultaneously released gamma rays are registered as coincidences by external detectors placed around the subject providing good sensitivity for the detection of the biotracer with a resolution within the range of 1 to 2 mm.

SPECT systems use radiopharmaceuticals that have a longer half-life and are widely used in clinics, being more readily available and less costly than PET tracers, which require a cyclotron for their production [21]. Nevertheless, the high sensitivity of PET tracers and their integration as biomarkers with multi-capability applications make them very well suited for small animal imaging and tracers. Examples of currently used radiomarkers are ^18^ F-FDG, an analogue of glucose, and ^18^ F-FLT, a thymidine analogue, which are extensively used as a biomarker of tissue metabolic activity and inflammation and cell proliferation, respectively. These techniques provide great potential to investigate physiological functions, track metabolic processes and quantify receptor density.

The main limitations of the SPECT preclinical systems include lower sensitivity and spatial resolution. The PET systems provide higher sensitivity, with a greater ability to quantify the tissue concentration of the tracer; however, the tracers have very short half-life and, as with the SPECT systems, do not provide anatomical information of the tissue/organ images. The advantage of both techniques is the high sensitivity in biological systems down to the picomolar range.

Imaging acquisitions may take up to 2 h, requiring adequate physiological monitoring to maintain good homeostasis which is crucial for the assessment of functional/metabolic parameters measured through the radiotracers. Temperature monitoring remains critical during PET/SPECT acquisitions as the body temperature strongly confounds the body’s metabolic functions. It is important to note that vascular access (e.g. cannulation tail vein) is required to inject the tracer through the general circulation to ensure its distribution through the tissues/organs during imaging. Furthermore, to investigate the kinetics of the tracer uptake, serial blood sampling is required for extracting an accurate arterial input function of the tracer used and also for assessing the integrity of the parent radiolabelled tracer. Such measurement poses a significant challenge in mice because blood volume is smaller and the heart rate is much higher in mice than in larger animals or humans. Several techniques have been proposed such as the use of beta-probe measurements, microblood sampling, arterial-venous shunts, factor analysis of dynamic structures and image-based measurement from a left ventricular region of interest [22]. The arterial blood sampling in small amounts at timed intervals is often considered the reference standard for true blood activity determination.

Animals may get exposed to radiation when imaged with PET or SPECT. The radiation exposure will depend on the dosage of the tracer injected. The tissue activity concentration in laboratory rodents is relatively higher than in humans, resulting in higher radiation dose. However, the large surface area compared to the small body volume of the animals allows for a larger escape of radiation. PET tracer doses typically range from 18.5 to 74 MBq (0.5 to 2.0 mCi) for rats and 1.85 to 7.4 MBq (50 to 200 μCi) for mice. There is a large variation in organ exposure related to the radionuclide, biodistribution and clearance of the compound.

Preclinical optical imaging includes bioluminescence and fluorescence imaging [23]. Fluorescence imaging uses external dyes or fluorescent markers which emit photons after excitation. Fluorescent probes are extensively used and can be found expressed in reporter proteins (GFP, RFP), in microspheres or as dyes that are widely used for labelling cells or for monitoring gene expressions. Bioluminescence relies on the production and emission of light that resulted from an enzymatic reaction between the luciferase enzyme and its substrate, luciferin, to produce light. The principal advantage of *in vivo* bioluminescence is that the equipment is highly sensitive and allows the direct detection of low emissions of light, with very little background, yielding a high signal-to-noise ratio. However, the technique requires the animal to be injected with the substrate. Fluorescence imaging involves no additional animal treatment but instead requires an external excitation source and multiple filters to obtain a spectrum of the light emitted which can compromise the sensitivity as there is a relatively high level of background-emitted light. Reduction of the autofluorescence is important to enable detection of weak fluorescent signals, increasing signal sensitivity. This is generally achieved by minimising skin fluorescence using nude animals, avoiding pigmented rodents and removing the fur around the area of interest. Recently, this has been minimised by the use of infrared spectrum fluorophores that guarantees acquisition with less background autofluorescence and also greater tissue penetration and sensitivity. These techniques are extensively used for monitoring the expression of transgenes, tumour growth and metastasis, infections and gene therapy assessments.

The advantage of these technologies is that they use low-energy, non-ionising radiation with high sensitivity down to microns and subpicomolar concentrations. Moreover, the acquisition times are short, thus minimising the time that animals are maintained under anaesthesia. The main challenges rely on the properties of the light sources, their wavelength, diffusion and absorption, which influence the penetration depth of the light into tissues, as well as the resolution. Also, this technology has no direct equivalent in the clinics, which compromises the translation of preclinical data.

High frequency ultrasound is based on the propagation of sound waves through the soft tissues that are being imaged. The preclinical systems are dedicated ultrasound systems that use high frequency modes (20 to 50 MHz) to provide good spatial resolution and adequate penetration for anatomical, functional and hemodynamic real-time information on preclinical models. These systems are relatively inexpensive and provide real-time imaging but are limited by the tissue depth (maximum penetration is around 15 mm) and the structures (artefacts caused by bone or air) that can be reached and visualised. This technique is extensively used in cardiovascular research, prenatal development and for monitoring tumour growth and metastasis progression. Acquisitions are generally quick, but it is important to continue monitoring body temperature, especially in rodents, to minimise any confounding effects on the cardiovascular function.

### Animal preparation for imaging procedures

Different procedures will be required depending on the imaging modality that will be used, the tissue/organs to be imaged and the *in vivo* experimental protocol required (e.g. fasting, artery/vein cannulation, ECG probes, tracheal intubation). All these procedures will influence the physiological parameters in the animals and will have to be accounted for when carrying out *in vivo* imaging.

#### Transport of animals and acclimatisation

Animals will have to be transported to the imaging facility. Such facilities should have an animal housing area to allow for the acclimatisation of the animals before imaging and also for monitoring their recovery after the experimental procedure. Not all the facilities are necessarily fully integrated within a centralised animal unit, and therefore, it is important that researchers are aware of the requirements for transporting the animals within the imaging facilities. These include (1) health screenings to minimise potential disease transfer and (2) acclimatisation periods to reduce transport stress-related responses for the animals. Furthermore, it is also important that animals are housed in an area near but separated from a procedure room in which all the animal preparation for imaging (e.g. vein cannulation) is carried out, as recommended by many codes of practice for the housing and care of animals used in research.

The standard recovery period after transport for rodents varies between 2 and 7 days, depending on the procedure and transport period. In general, it has been reported that there was an increase of glucocorticoid concentrations for up to 2 days, decrease in body weight and immune response suppression of up to 48 h after transport in rodents [24]. Similarly, a recovery period should also be considered after imaging, depending on the type of anaesthetic, the length of anaesthesia and experimental procedures carried out (e.g. significant blood volume withdrawal, surgically induced procedure, bone marrow reconstitution).

#### Health checks

The health and general conditions of the animal will affect its physiological response to the anaesthesia and the outcome of the imaging procedures. Researchers must be aware that rodents are prey species, so they may hide or compensate for distress and/or disease conditions to such extent as they appear to be healthily normal. It is important that, prior to imaging and induction of anaesthesia, animals are carefully evaluated for good health. Wherever possible, within the constraints of the study, animals in good general condition should be used. This is also important for good experimental study design as it minimises the variation between individuals, leading to more accurate, reproducible results and thus reduction in animal numbers used.

The pre-anaesthetic exam should contain but is not limited to confirmation of animal’s identification, sex, age, body weight, body condition, skin condition, estimate hydration, colour of mucous membranes, heart rate and rhythm, respiratory rate, signs of diarrhoea, body temperature, evidence of normal food and water consumption and normal production of urine and faeces in the cage. Overall, the body condition of the animal is very important to anaesthesia induction, maintenance and recovery (e.g. very obese animals may respond slowly to drugs).

#### Food withdrawal

**Figure 1:**
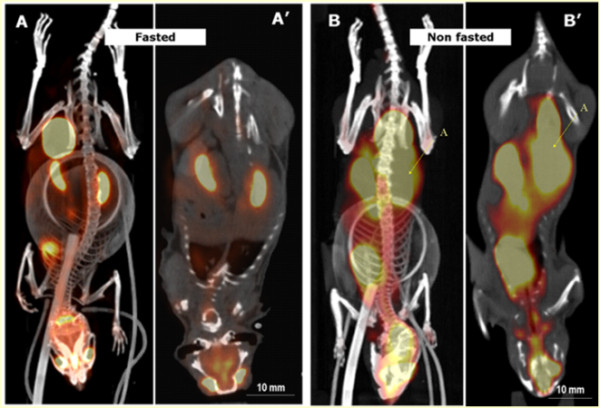
** Example of PET/CT acquisitions showing fasting effects on biodistribution of 18 F-FDG-PET tracer in C57BL/6 mice.** (**A**, **B**) Maximum intensity projection, and (**A′**, **B′**) two-dimensional coronal view. The fasted animals displayed a more targeted and selective uptake of the glucose analogue tracer throughout the body, avoiding the general uptake throughout the whole digestive system due to food ingestion.

Rodents do not require compulsory fasting prior to anaesthesia as they do not possess a vomiting reflex. However, fasting may be required for some imaging procedures. Fasting can be an effective way to ensure uniformity of PET imaging with ^18^ F-FDG (Figure 1), by decreasing the blood glucose levels associated with food intake and thus allowing for a more controlled assessment of metabolic rate of glucose throughout the body. In rodents, fasting times of up to 6 h are effective to clear the stomach food [25], but longer fasting periods will have important detrimental side effects in the animal conditions including loss of body mass and decrease of blood glucose and fatty acid levels [26]. Generally, researchers may fast the animals overnight, removing the food on the evening prior to the imaging day. However, Levine and Saltzmann [27] observed loss of body weight, depletion of liver glycogen, decrease in blood glucose and loss of amino acids in rats that were starved overnight and reported that feeding sugar overnight maintained metabolic homeostasis in rats and is preferable to overnight starvation. If anaesthesia is required for a pregnant animal, the need to withhold food should be carefully evaluated.

Rodent food consumption is associated with the animals’ circadian cycle, and thus, the removal of food overnight (dark hours) will have a stronger impact on their caloric intake as compared with that for light hours. It is important to control the length and timing of fasting before imaging studies and to ensure that experimental protocols are well standardised to ensure the reproducibility between imaging studies. Similarly, it is also very important to consider the effects of fasting if any specific dietary requirements are being used and how this can influence the outcome of the imaging studies (e.g. some diets can induce strong background autofluorescence in mice [28]).

#### Premedication

Premedication refers to the administration of any drugs in the period prior to induction of anaesthesia. A wide variety of drugs can be used during the preparation of animals for imaging studies include the following: anti-cholinergic drugs which are used to decrease oral and respiratory secretions, maintain the heart rate and decrease gut motility; tranquillisers which are used to relieve anxiety, produce calmness, aid restraint, assist with a calm postoperative recovery and reduce dose of anaesthetic needed; narcotics which are used to sedate an animal. They reduce the amount of anaesthesia needed, induce smooth recovery and provide postoperative pain relief. Other analgesics that may be given are non-steroidal anti-inflammatory drugs, such as carprofen, to provide pain relief for up to 24 h after the procedure if minor surgery has been performed.

### Anaesthesia

Anaesthesia is generally required during imaging experiments in order to ensure humane restrain of the animals. Anaesthesia is described as a state of lack of awareness of a part or the whole body of the animal induced by the administration of specific drugs that depress nervous tissue activity. It involves a triad effect including analgesia (pain relief), loss of consciousness and immobilisation (muscle relaxation) [29]. While most of the imaging procedures are non-invasive, it is very important to ensure that anaesthetised animals remain in a stable physiological state with consistent cardiovascular output, body temperature and blood oxygenation. Indeed, many studies (e.g. functional MRI) are highly sensitive to these parameters. Therefore, it is very important to address the physiological effects that different anaesthetic regimes are likely to have during imaging.

In order to achieve the effects sought for anaesthesia in rodents, there is an inevitable autonomic nervous system depression which induces cardiovascular depression (e.g. reduction in cardiac output, blood pressure, altered blood flow) and respiratory depression (e.g. hypoxia, hypercapnia, related acidosis) and induces hypothermia, affecting body metabolism. Moreover, rodents are prey species with small body size, high body surface/weight ratio and high metabolic rate which compromises the pharmacological efficacy of injectable agents and their body temperature regulation. Consequently, high doses of agents are required to induce unconsciousness, which has also a detrimental effect on autonomic depressions. In this vein, the use of inhalation anaesthesia in rodents allows these adverse effects to be more controlled due to their rapid onset and recovery time and low metabolism. Hence, it is highly recommended to use inhalation anaesthesia for imaging purposes.

It is also important to appreciate the variation in response to anaesthetics between different animal strains. Therefore, it is important to reassure and adjust the anaesthesia protocol to the particular needs of the strain and experimental set-up.

#### Anaesthetic regimes and equipment

Injectable and inhaled anaesthetics are commonly used in rodents. The latter method is most suitable for imaging; due to its rapid onset and recovery times, it allows a much greater control of the anaesthetic doses and maintenance times of anaesthesia than when using injectables. Moreover, inhaled agents are eliminated quicker via the lungs, whereas injectable agents need to be metabolised by the liver and excreted by the kidneys [30]. This allows the animals to recover quicker, which is important in regaining normal physiology to control post-operative hypothermia and fluid or electrolyte imbalance. Some of the newer injectable agents have specific reversal agents which speed recovery and help to overcome many of these potential problems.

If injectable agents are used for long-term anaesthesia, as can be the case in MRI acquisitions, there is an increasing risk of developing hypoxia, especially if oxygen supplementation is not provided, which could lead to respiratory depression and hypercapnia and acidosis if prolonged.

*Injectable anaesthetics*. Anaesthetic dose rates for injectable agents will depend on species used, administration route, age, sex, strain, body condition, environment, experimental set-up, previous drug treatments and the level of anaesthesia required. During the initial period of use, it is important to monitor animals closely and make any adjustments necessary for future use.

Increasing the length of anaesthesia with a given drug may require a second or further injection at intervals during the procedure. However, increasing the initial dose will result in an increase in depth at the time of peak action with the risk of reaching a point of an overdose. Also, giving intermittent top-up doses of the drug will cause the depth of anaesthesia to vary considerably. This may be overcome by administering it as a continuous infusion so that steady plasma concentrations of the anaesthetic are maintained. However, this can be challenging depending on the pharmacodynamics of the anaesthetic agent and the experimental set-up. In general, repeated doses will have progressively greater effect and, in addition to extending the duration of surgical anaesthesia, can prolong the sleeping/recovery time following anaesthesia. This is particularly critical when using opioids. If the animal does eventually wake up, the residual effects of the drug may persist for a long period of time [31].

The two anaesthesia combinations that are widely used in laboratory rodents are fentanyl-based and ketamine-based. Agents available are as follows:

1. *Fentanyl/fluanisone (Hypnorm™; mostly in combination with benzodiazepine)*. Hypnorm™ (tradename) consists of two components: fentanyl and fluanisone. Fentanyl is a potent, short-term, opioid agonist analgesic. Fluanisone is a butyrophenone tranquilliser and is used to suppress some of the undesirable effects of the narcotic (fentanyl) such as vomiting and excitement. This neuroleptanalgesic combination induces moderate respiratory depression and a poor degree of muscle relaxation. Hypotension and bradycardia may also be noted.

Fentanyl/fluanisone (Hypnorm™, Vetapharma, Leeds, UK) is licensed for sedation in rodents, rabbits and guinea pigs. As there is poor induction of muscle relaxation, it cannot be used on its own for surgical procedures. Fentanyl/fluanisone in combination with benzodiazepine (midazolam or diazepam) is licensed for surgical anaesthesia in rodents, rabbits and guinea pigs. This combination reduces the dose of Hypnorm™ by 50% to 70%, and benzodiazepine produces good muscle relaxation. The most commonly used benzodiazepine tranquilliser is midazolam (Hypnovel™) as it is water soluble.

Depending on the animal’s depth and length of anaesthesia, a ‘top-up’ administration may be necessary. While the effects of midazolam or diazepam can last for several hours, anaesthetic duration is lengthened in the first instance by giving additional Hypnorm™. However, due to enterohepatic recirculation of the metabolised anaesthetic products, a relapse of the anaesthesia effects may occur, which means that Hypnorm™/Hypnovel™ combinations can have a prolonged recovery time or ‘sleep’ time (average >4 h for rodents). Monitoring must continue during this period; the body temperature, maintained. This combination also appears to sensitise rodents to auditory stimuli.

2. *Ketamine*. Ketamine is one of the most commonly used anaesthetics in the veterinary field, and the commercially available injectable versions of ketamine hydrochloride are known as Vetalar™ or Ketaset™. Ketamine produces a state of ‘dissociative anaesthesia’ in which there is profound analgesia, light sedation and muscle rigidity (stage of catalepsy). It does not depress the central nervous system (CNS), and thus, reflexes remain intact. Some side effects are as follows: eyes remain open, and ophthalmic ointment is recommended; presence of spontaneous movements and muscles are tense which causes an initial increase in blood pressure although there is also a peripheral vasoconstriction. In contrast to other anaesthetics, ketamine does not depress respiration or cardiac output [32].

Used on its own, ketamine does not achieve surgical anaesthesia but is extremely useful when administered in combination with xylazine, medetomidine or diazepam for the production of surgical anaesthesia. Ketamine is contraindicated for use in animals with renal or hepatic disease [33]. During ketamine recovery, animals are hyper-responsive and ataxic. Recovery may also be associated with behaviour alterations [34]. Combinations of ketamine for general anaesthesia are as follows:

a) *Ketamine + alpha-2 adrenergic agonist sedatives (alpha 2s): medetomidine (Domitor®, Pfizer Animal Health, Tadworth, UK) or xylazine (Rompun®, Animal Bayer Health, Newbury, UK)*. Ketamine/alpha-2 combinations are suitable for surgical anaesthesia but are significantly hypotensive and can induce profound bradycardia and respiratory depression. These combinations provide, on average, 30 min of surgical anaesthesia. The sedative can be reversed using the alpha-2 antagonist atipamezole (Antisedan®, Pfizer Animal Health), but this should not be given until the ketamine has worn off, at least 30 min after injection. Atipamezole is a highly specific antagonist for medetomidine but has some effect in reversing xylazine as well. Note that the combination of ketamine/medetomidine + buprenorphine as preoperative analgesia has proved toxic in rats [35], and caution is advised when considering this regime, but this is strain dependent. This adverse effect has not been observed when buprenorphine is administered post operatively. Ketamine-xylazine combinations may affect the brain haemodynamics, causing a reduction of the cerebral blood flow and affecting brain oxygenation which can have confounding effects on nuclear magnetic resonance perfusion imaging [36]. Also, ketamine combinations with xylazine have substantial cardiovascular effects, manifested by low pulse rates and hypotension [37].

b) *Ketamine and benzodiazepine combinations*. Ketamine can also be combined with benzodiazepines, such as midazolam, to provide 20 to 30 min of light anaesthesia in rodents. The depth of anaesthesia may only be sufficient to permit minor procedures to be performed but could be used for immobilisation. The degree of respiratory and cardiovascular depression is less than when ketamine is combined with an alpha-2 agonist.

3. *Alfaxalone (Alfaxan)*. Alfaxalone (Alfaxan® Vetoquinol UK Ltd, Buckingham, UK) is an anaesthetic steroid. The fast metabolism of this drug enables it to be used for long periods of anaesthesia by continuous infusion in rodents and maintain a fairly rapid recovery following the last administration, but it has to be administered intravenously (i.v.) in rodents [38]. Renal and hepatic perfusion and respiratory function is well maintained.

4. *Propofol (Rapinovet®, Diprivan®)*. Propofol is an isopropylphenyl compound that is available for intravenous use as a 1% solution in soybean oil, glycerol and egg phosphatide. Propofol exerts its CNS effects via modulation of the gamma-aminobutyric acid (GABA) channels through different sites than the barbiturates, steroids or benzodiazepines. It rapidly induces unconsciousness; recovery is more rapid and complete with minimal residual CNS effects compared with thiopentone, holding good potential as an anaesthetic regime for functional MRI (fMRI) studies [39]. Its rapid elimination from the body and lack of cumulative effects make it particularly suitable for continuous infusion, either alone for restraint or in combination (e.g. ketamine, an opioid or isoflurane) for surgical anaesthesia. However, it does not induce analgesia, and thus, analgesics should be considered when painful manipulations or procedures are performed. Cerebral blood flow, perfusion pressure and intracranial pressure decrease following propofol administration [40]. It is a potent respiratory depressant, and apnoea is common on induction unless the drug is given slowly.

5. *Barbiturates*. Barbiturates modulate GABA transmission, the most common inhibitory transmitter in the mammalian nervous system, inducing depression activity in the reticular formation (necessary for maintenance of wakefulness). In addition, barbiturates selectively depress transmission at the sympathetic ganglia, which may contribute to decreased blood pressure following their administration. These agents are highly metabolised by the liver, and metabolites accumulate in the body over time. Many of these agents will produce sedation associated with severe respiratory depression and general anaesthesia as larger doses are administered. At high doses, these agents are utilised for euthanasia. Some barbiturates are caustic substances (high alkaline pH) when injected into the living tissue and so must be given intravenously or diluted and given intraperitoneally (i.p.) (only for pentobarbitone). Subcutaneous or intra-muscular routes should be avoided:

a) Pentobarbitone. This is a barbiturate with a medium length of action that can be given i.p. or i.v. in a range of species. Its main effect is one of hypnosis, with poor analgesia and muscle relaxation except at near lethal dose rates. There is profound cardiovascular and respiratory depression particularly in rats. As pentobarbitone is highly metabolised by the liver and has cumulative effects, it is not ideal for long-term anaesthesia.

b) Thiopentone and methohexitone. Short-acting barbiturates are commonly given intravenously; they have poor analgesic effects yet reasonable muscle relaxation. Respiratory function is depressed and may be temporarily suspended during induction. Recovery from single doses of thiopentone and methohexital is due to redistribution of the drug from brain to non-nervous tissues (fat, primarily viscera and skeletal muscle). In the case of methohexital, rapid hepatic metabolism contributes remarkably to recovery. It induces a transient small decrease in arterial blood pressure that is compensated for by an increase in heart rate. Myocardial depression is minimal and far less than would occur with volatile inhalants. Thiopental anaesthesia has minimal effect on the neurochemical profile in the rat brain but substantially increases brain glucose content in the cortex as detected by *in vivo* 1H MRS [41]. Thiopentone acts as an irritant outside the vein and can induce perivascular necrosis. Methohexitone has a shorter half-life and a rapid metabolism, so it is suitable for long-term infusion. It is not an irritant outside the vein, but tremors can occur if given without premedication.

6. *Miscellaneous*. The following are the miscellaneous agents:

a) Chloral derivatives (e.g. chloral hydrate, α-chloralose)

· *Chloral hydrate* is a reliable sedative hypnotic, but it has poor analgesic properties. Consequently, it has frequently been used in combination with some other drugs such as magnesium sulphate when general anaesthesia was the desired endpoint [42]. Chloral hydrate is medium acting (1 to 2 h). Intraperitoneal administration of chloral hydrate to rats is associated with paralytic ileus. For this reason, it is recommended that this agent be only used for non-recovery procedures [43].

· *α-Chloralose* is a hypnotic frequently used in neuroscience experiments [44]. This drug has no analgesic activity but can be combined with local or regional anaesthesia (novocaine, marcaine, bupivicaine), to produce sufficient tranquilisation and anaesthesia to allow minor surgical procedures. The duration of action of α-chloralose is prolonged (8 to 10 h), making it a possible choice for long-term anaesthesia.

Since this drug is probably a hypnotic rather than a true anaesthetic, with unproven analgesic potency, it appears that the most ethically and scientifically acceptable use of this agent in rodents is to provide long-lasting anaesthesia for procedures involving no painful surgical intervention. The primary advantage of this drug is the minimal cardiopulmonary depression seen at the normal doses (high doses can cause severe respiratory depression). However, it is very irritating to the GI tract, causing a dynamic ileus if given i.p. and ulcers if given orally. Therefore i.v. use is the only route recommended. This drug should not be used if any other alternative is available. α-chloralose has been used for fMRI studies providing consistent and reproducible blood oxygen level dependent (BOLD) signals, but it is important to monitor the animal closely during recovery of anaesthesia [45].

b) Urethane. Urethane produces long periods (8 to 10 h) of anaesthesia, has a wide safety margin and has little effect on normal blood pressure and respiration. It produces sufficient analgesia to allow surgical manipulations [46]. However, the drug should be handled with ‘extreme care’ as it is considered to be cytotoxic, carcinogenic and immunosuppressive. Due to the potential hazards to staff, other anaesthetics should be assessed and an alternative regimen used wherever possible. Because of its carcinogenic effects urethane should only be used in animals that will not recover following anaesthesia.

c) Tribromoethanol (Avertin®). Tribromoethanol is widely used as a short anaesthetic agent for embryo-transfer surgery for the generation of transgenic mice [47]. Potential side effects such as local irritation, fibrous adhesions in the abdominal cavity and mortalities have been reported. Tribromoethanol has many features of a good anaesthetic: a wide margin of safety, rapid induction and recovery, simple route of administration (i.p.), provides good muscle relaxation and loss of reflex activity. However, there have been conflicting reports of toxic side effects [48]. See Table 1 for summary of properties of injectable anaesthetics.

*Inhalation anaesthesia*. Inhalation anaesthesia is the method of choice for general imaging protocols for laboratory rodents. Highly volatile anaesthetic agents are transported through a carrier gas to the animals via a breathing circuit with an integrated vaporizer which allows regulation of the anaesthetic concentration and flow into the animal.

Oxygen is most commonly used as the carrier gas for the inhalation anaesthetic agent. It is supplied in pressurised cylinders and delivered via a pressure regulator to a flow metre that provides adequate flow rates (0.5 to 1.5 l/min) through an integrated vaporiser. The lung capacity of the animal and to a lesser extent the efficiency of the anaesthesia circuit used will determine the gas flow rate needed. The circuit is also necessary to remove exhaled gases and to provide a method for assisting or controlling ventilation. Non-rebreathing circuits are usually used in laboratory animals to ensure minimum dead space and resistance. Also, they provide a known inspired concentration as the fresh gas inlet goes directly to the animal, allowing for a good regulation of the anaesthesia concentration in the inspired gases. The most commonly used anaesthetic circuits for rodents are the Bain’s co-axial T-piece facemask in which the fresh gas inflow pipe runs inside the reservoir limb and the open facemask system. One of the drawbacks of the latter is that removal of waste anaesthetic gases is difficult because gas escapes all around the mask, and this could result in serious health and safety issues for the operator.

· *Inhalation anaesthetic agents*. Inhaled anaesthetics are volatile compounds that have specific effects on the CNS. Different inhalation anaesthetics require different concentrations to induce and maintain anaesthesia, each agent differing in potency and efficacy. The desirable inhalant must produce complete anaesthesia, provide a rapid induction and recovery and be safe, non-irritant and non-explosive.

1. *Isoflurane and halothane.* These are the most commonly used inhalation anaesthetics in laboratory animals. These agents are broadly similar in speed of onset/offset and potency (Table 2). Isoflurane is extensively used due to the fact that it is minimally metabolised (<0.17%) by the liver and therefore is less toxic to the animal’s metabolism as compared with injectable anaesthetics. However, both agents can be rapidly fatal if overdosed, and therefore, animals should be individually induced and transferred to a lower maintenance percentage immediately once they are unconscious, i.e. have lost their righting reflex.

**Table 1:** Properties of main anaesthetic agents used in preclinical research: halothane and isoflurane

**Agents**	**Advantages**	**Disadvantages**	**Dosing**
Halothane	Potent anaesthetic	Highly metabolised (hepatotoxic)	Induction 3% to 4% Maintenance 1% to 2% (rats and mice)
	High therapeutic index	Cardiovascular depressant	
	Rapid induction and recovery (1 to 3 min)	Moderate hypotension: reduction in cardiac output and peripheral vasodilatation)	
	Adequate muscle relaxation	Respiratory depressant	
	Non-irritant, non-flammable nor explosive	Halothane sensitises the heart to catecholamines (sympathetic stimulation)	
	Easy to vaporise		
Isoflurane	Similar physical properties to halothane	Decreases arterial blood pressure (vasodilatation)	Induction 3% to 4% (rats and mice) Maintenance: 1.5% to 2% (mice)1.5% to 2.5% (rats)
	Rapid induction and recovery	More expensive than halothane	
	Low toxicity and metabolic activity: highly safe	Strong smell: aversive	
	Suitable for high frequency and long-term anaesthesia	More potent respiratory depressant than halothane	
	Minimal cardiovascular depression		
	Moderate respiratory depression		
	Good muscle relaxation		

Isoflurane itself has become a preferred general anaesthetic agent for cardiovascular studies because it causes less depression of cardiac function than injectable general anaesthetics [49]. However, it decreases blood pressure by reducing peripheral resistance [50]; see Table 2 for further properties.

**Table 2:** Summary of the properties of injectable anaesthetics

**Anaesthetic agent**	**Advantages**	**Disadvantages**	**Dosing**
Fentanyl/fluanisone (Hypnorm™)-based combination	Good analgesic effect	Cardiovascular and respiratory depression	10 ml/kg (mouse), 2.7 ml/kg (rat) i.p. mixture Hypnorm™/Hypnovel™ (midazolam)/water mixture (1:1:2 volume) (120 to 140 min sleep time)Hypnorm™ top-up 0.3 ml/kg (mouse), 0.1ml/kg (rat) i.p. (30 to 40 min sleep time)
	Sedative	Poor muscle relaxation alone	
	Possible to top-up for long-term anaesthesia	Risk of enterohepatic recirculation: relapse	
	Reversal of sedative effect with buprenorphine to speeds up recovery time	Prolonged recovery time	
		Hypersensitivity to noise	
Ketamine-based combination	Analgesic effects	Muscle rigidity +++ unless combined with other agents.	Ketamine + medetomidine 75 mg/kg + 0.5 to 1 mg/kg i.p.(Ketamine + xylazine 75 to 100 mg/kg)/(10mg/kg i.p.)(60 to 120 min sleep time ) (mouse and rats)Atipamezole 1mg/kg i.p.
	Light sedation	Increases intracranial pressure.	
	Wide safety margin	Recovery often involved with ataxia and hyper responsiveness.	
		Can increase blood pressure	
Alfaxalone (Alfaxan)	Minimal respiratory or CVS depression	Administration route i.v. (rodents, cats) or i.m. (primates)	15 to 20mg/kg (mouse), 10 to 12mg/kg (rat) i.v.(10 to 15 min sleep time after bolus)0.25 to 0.75 mg/kg/min i.v. infusion (long term)
	Rapidly metabolised, repeated doses do not accumulate.		
	Suitable for long-term anaesthesia in rodents		
Propofol (Rapinovet®, Diprivan®)	Rapidly metabolised, continuous infusion possible for long-term anaesthesia.	i.v. use only	26 mg/kg (mouse), 10 to 12mg/kg (rat) i.v.(10 to 15 min sleep time after bolus)2 to 2.5 mg/kg/min i.v. infusion (long-term, mouse) 0.5 to 1 mg/kg/min i.v. infusion (long-term, rat)
		No analgesic properties	
	Rapid recovery	Severe respiratory depression	
	Can be used in animals with hepatic or renal impairment.	Apnoea can occur after i.v. bolus	
Barbiturates products	Sedative effect	No analgesic properties	Pentobarbitone, 40 to 50mg/kg i.p. (mouse) (120 to 180 min sleep)
	Good hypnotic effect	Severe respiratory depression and hypotensive	
	Reasonable muscle relaxation	Easy to overdose	Thiopentone, 30 mg/kg i.v. 15 min sleep (rat)
		Metabolites accumulate with time	
		Caustic substances (thiopentone only i.v. route)	
Chloral hydrate	Sedative effect	No analgesic properties	300 to 400 mg/kg i.p. (1 to 2 h sleep time) (mouse and rats)
	Good hypnotic effect	Paralytic ileus noted in rats	
	Minimal CVS and respiratory depression	Terminal/non-recovery work only	
*α*-Chloralose	Sedative effect	No analgesic properties	50 to 60 mg/kg i.p. (rats), 120 mg/kg i.p. (mouse)(8 to 12 h for non-recovery only)50 mg/kg i.v. bolus followed by 25 to 40 mg/kg/h (rats)
	Good hypnotic effect	i.v. use only	
	Suitable for long-term anaesthesia	Slow induction and recovery associated with involuntary excitement	
	Minimal CVS and respiratory depression	Terminal/non-recovery work only	
Urethane	Suitable for long-term anaesthesia.	Carcinogenic: only allowed to be used with special justification	0.8 to 1.3 g/kg i.p. (mouse and rats)Duration of action 8 to 10 h (non-recovery only)
	Minimal CVS and respiratory depression	Terminal/non-recovery work only	
			
Avertin® (tribromoethanol)	Wide safety margin	Local irritation/peritonitis	0.015 ml/g body wt of 2.5% i.p.
	Good muscle relaxation	Handling and storage safety issues	30 min; supplemental doses of anaesthesia: minimum of 1/2 of the initial dose up to 1 ml maximum volume per animal
	Rapid induction and recovery	Toxic effects	

2. *Enflurane, desflurane (I653) and sevoflurane* are all used in human anaesthesia; some offer no significant advantages over isoflurane for the majority of procedures while being considerably more expensive.

Sevoflurane was first synthesised in the late 1960s but was not developed for approval until 1990 in Japan because of concerns about degradation with soda lime (to produce compound A, which can produce renal toxicity) and release of fluoride ion during metabolism (which can also produce nephrotoxicity). Neither of these concerns has been proven to be clinically important in the human or veterinary field. It is less soluble than isoflurane, which means that inductions and recoveries are even faster. Sevoflurane also appears to be better tolerated by face mask and chamber induction because it has low pungency and low airway irritability. Since sevoflurane is less potent than isoflurane (minimum alveolar concentration for sevoflurane is 2.4% for dogs), the doses used are usually higher than those used for isoflurane. However, there have been recent reports that isoflurane and sevoflurane provided an equally reliable anaesthesia in laboratory mice [51].

3. *Nitrous oxide*. Nitrous oxide (N_2_O) is not potent enough for induction of anaesthesia and is, therefore, unsuitable for use alone. It is an analgesic, relaxant and, to a certain extent, tranquilliser. Nitrous oxide has minimal cardiovascular and respiratory effects. It is generally used in combination with other volatile agents to reduce the required concentration of that agent, allowing for reduction in dose of other agents, reducing costs, speeding up induction and providing additional muscle relaxation. However, it is important to be aware that the oxygen percentage of the inspired gas is lowered, and there is a risk for ‘second gas effect’, in which after prolonged anaesthesia, oxygen can be displaced from the lungs by the nitrous oxide as it is breathed off, causing diffusion hypoxia and can lead to suffocation. It is important to supplement with 100% oxygen after N_2_O is discontinued to protect against diffusion hypoxia.

4. *Diethyl ether.* Diethyl ether is occasionally used in laboratory animals. It is a good analgesia and promotes muscle relaxation with minimal respiratory and cardiovascular depression. However, it is flammable, explosive and aversive, causing nauseas and vomiting. Most units have prohibited the use of this agent due to serious health and safety concerns and the fact that superior anaesthetic agents are now available.

5. *Methoxyflurane*. Methoxyflurane is the least volatile and the most metabolised of the inhalants. It produces very slow induction and recovery and is used occasionally in laboratory animals (e.g. useful for neonatal rodents).

### Monitoring anaesthesia during imaging procedures

While the ideal anaesthetic agent would produce reversible unconsciousness and analgesia with no other homeostatic effects, the reality is that all anaesthetic drugs affect other body systems to some extent. The most important effect of anaesthetic drugs is depression of respiration, the cardiovascular system and thermoregulation. Therefore, one must seriously assess the therapeutic dose required for anaesthesia, what the likelihood is to affect image acquisitions and the outcome of scientific data acquisition interference during the procedure. It is also important to clearly understand that extreme deviations from maintaining normal physiology during anaesthesia can be life threatening for the animal.

While it is not clearly defined how frequently one should monitor the animals during anaesthesia, it is obvious that the more invasive the procedure and/or the longer time under anaesthesia, the more likely it is to interfere with normal homeostasis and thus greater the need for monitoring. A general approach is to continuously monitor cardiovascular and respiratory function as the minimum standard applicable to veterinary anaesthesia. Indeed, for laboratory animals, this may require specialised monitoring equipment.

The position of the animals is also of importance as physiological parameters can be quite affected by the bed systems used in different imaging modalities. It is crucial that the neck and head are well positioned with the airway maintained well and open at all times, without restricting breathing or/and cardiovascular function, e.g. to minimise pressing equipment on the thoracic cavity. Body and extremities should be positioned without restricting the circulation and avoiding any bruising, strains or avulsion in the body structures.

Generally, the monitoring procedure should cover the four body systems, namely central and peripheral nervous, respiratory, cardiovascular and musculoskeletal systems. Basic parameters should include the following:

a. Respiratory rate, depth and character

b. Heart rate, rhythm and pulse intensity

c. Body temperature

d. Colour of mucous membrane, extremities and capillary refill time (peripheral perfusion)

e. Central nervous system (muscle tone and reflexes)

Direct access and visualisation of the animal while placed in the scanner during imaging can be challenging, especially for assessing parameters such as capillary refill and muscle tone and reflexes.

#### Respiratory system

**Figure 2:**
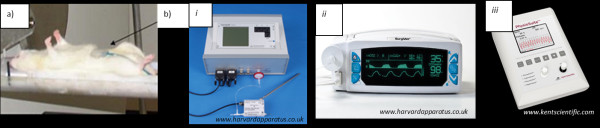
** Image displaying some of examples of respiratory monitoring equipment.** (**a**) Respiratory sensor (VioHealthcare, Uckfield, UK); (**b**) Capnographs used for rodents: (i) type 340 Capnograph system (Harvard Apparatus, Holliston, MA, USA) with specifications for working with mouse or rats; (ii) MRI adaptable system: V9004 Capnography/Pulse oximeter (Harvard Apparatus); and (iii) PhysioSuite (Kent Scientific Corporation, Torrington, CT, USA) CapnoScan to measure the end tidal CO_2_.

Respiratory monitoring can be based on clinical observations, including observation of the chest motion, or assessed through detection of breathing motion by compressions of a respiratory sensor placed in contact with the chest. Respiratory motion detection systems are extensively used during imaging as they are highly compatible with and safe to use for most of the imaging modalities (Figure 2).

Motion artefacts due to breathing which can seriously compromise imaging acquisitions can be eliminated from the acquired images by employing gating techniques, which allow the synchronisation of data collection with the respiratory cycle. Typically, the largest movement of the diaphragm and abdomen occur between inspiration and expiration; thus, selective acquisition during the still part of the respiratory cycle can be effective in reducing breathing artefacts.

Other advanced respiratory monitoring systems include digital systems that can monitor and detect the changes in gas temperature and/or compositions between inspiration and expiration at the endotracheal tube connector or face masks:

1. *Capnograph* measures the CO_2_ level through a highly sensitive infrared spectroscopy CO_2_ sensor in the inhaled and exhaled gas, based on the CO_2_ values in the venous return to the heart and the effectiveness of breathing. This equipment is a very good indicator of the respiratory function, providing continuous measurements of the CO_2_ levels. The CO_2_ levels rise rapidly during the first part of exhalation and then flatten off. If the pulmonary function is compromised or there is poor lung perfusion, the flatten-out phase will disappear. The level of CO_2_ at the end of expiration (etCO_2_) is normally within a few millimetres of mercury of the arterial CO_2_, and it is very useful for assessing adequacy of ventilation during both spontaneous and artificial ventilation [52].

One problem with this equipment is that the gas sampling rate can be quite fast and removes excessive amount of gas from the airway. There are some adapted capnographs for rodents that utilise small volumes and are equipped with a pressure sensor for measuring tracheal pressure and a pneumotach with differential pressure transducer for measuring respiratory airflow. These sensors are mounted directly on the tracheal or intubation cannula and connected to the control unit to minimise the dead space. The tracheal pressure and the respiratory flow are important signals to monitor mechanical ventilation when using a respirator, aiding in avoidance of barotraumatic ventilation.

There are several capnographs with specifications for working with mouse or rats, but unfortunately, the equipment tend to be not MRI compatible. There are few MRI adaptable versatile capnographs on the market which can include pulse oximetry to measure PO_2_, PCO_2_ and fractional inspired oxygen measurements. (Figure 2).

2. *Blood gas analysis*. Arterial blood gas analysis is the gold standard for assessing respiratory function. The pH, PO_2_ and PCO_2_ levels of the blood sample are measured with specific electrodes and the bicarbonate concentration is also calculated. Through these measurements, we can obtain a detailed picture of the state of the respiratory system:

· PO_2_. It is standard for measuring blood oxygenation; decrease in PO_2_ is due to hypoventilation, inspiration of hypoxic gas mixtures or impaired gas exchange.

· PCO_2_. Levels of PCO_2_ are established by the balance between CO_2_ production and elimination. The PCO_2_ is fairly constant, and the arterial level is determined by elimination.

· HCO^3−^ concentration. Base excess and pH measurements provide information of acid and base balance. These measurements may be very relevant when imaging animals with metabolic disorders which may have acid and base disturbances.

**Figure 3:**
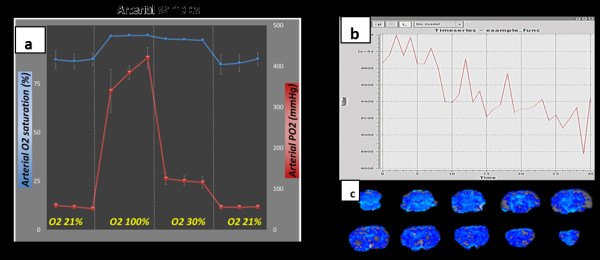
** Changes in arterial O**_**2**_**saturation and PO**_**2**_**and examples of whole pattern activation.** (**a**) Changes in arterial O_2_ saturation and PO_2_ under different O_2_ gas concentrations during anaesthesia. These parameters can affect the BOLD response during MRI; therefore, it is important to monitor blood gas levels during such functional MRI studies [54]. (**bc**) Example of whole pattern activation in a rat brain erratically induced by too deep isoflurane anaesthesia. Such a ‘bad’ activation pattern in the whole brain (c) would mask any specific activation due to a pharmacological and/or sensorial stimulus, and in this case, the time course shows (b) a general decline in signal, and no effect of the challenge is visible.

A blood gas analyser is not a routine piece of monitoring equipment, and interpretation of the results requires knowledge of pulmonary and renal physiology. The equipment needs to be near the imaging equipment, and in particular, for the rodents, one of the main limitations is the volume of blood that can be removed and the frequency of sampling [53]. However, the system provides invaluable monitoring data for animals used for cardiovascular and respiratory research. It is also standard to use it for functional MRI studies which are based on measuring changes in the blood oxygenation levels in microcirculation in the tissues [54,55] (Figure 3).

#### Cardiovascular system

Basic cardiovascular monitoring includes assessment of the following:

· *Heart rate*. This is generally assessed through palpation of the chest wall or auscultation using a stethoscope, but during imaging, the animal is generally placed within a restricted area with limited access (e.g. imaging platform). Hence, most of the modalities will have an electrocardiograph system implanted with specific electrodes adapted for each imaging modality.

· *Pulse.* The pulse is generally measured using a pressure cuff or an oximeter (see below).

· *Capillary refill time*. It is measured by applying pressure to a pink extremity or a mucous membrane (e.g. gums) and by checking the time for pinkness to re-invade the pressurised whitened areas (1 to 2 s).

· *Colour* of mucous membranes (gums, perianal area, conjunctiva) or hairless extremities.

**Figure 4:**
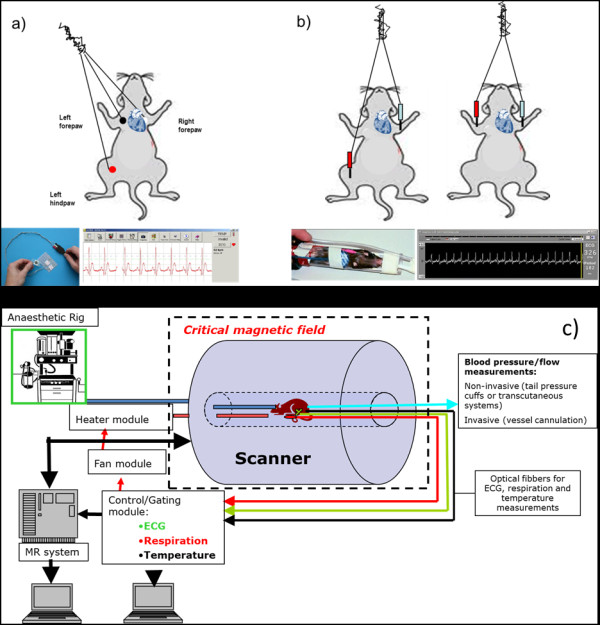
** ECG electrodes systems.** (**a**) System BioVet™ (©m2m Imaging Corp, Newark, USA): the carbon fibre electrodes are applied directly in contact with the cleaned and shaved chest skin and applied with gel electrode so that a minimal impedance electrical connection is made with the electrode. (**b**) Model 1025 small animal monitoring and gating system (Small Animal Instruments, Inc., Stony Brook, NY, USA): the ECG system used for the MRI scanners uses sub-dermal needle electrodes, pads or surface electrodes. The placement of the electrodes is typically in or on the right forepaw and the left hind pore, or electrodes are placed in the forepaw as long as it is across the heart plane. All the wire bundles within the scanner should be taped to eliminate unwanted movement from the gradient vibration and/or air flow. (**c**) Schematic representation of the fMRI setting: all the equipment needs to be non-ferromagnetic, and it is connected to a module system which allows gated acquisition of images, avoiding interferences from motion due to breathing and /or heart beating. Body temperature is also regulated through a heating module (small rodent heater system; Small Animal Instruments, Inc.) to monitor and control the animal temperature during imaging. The system software continuously processes the temperature measurements and sends an optical control signal to the heater control module. The rate of change of temperature is monitored, and heater control is adjusted to regulate temperature changes. Mouse temperature variations of less than ±0.1°C can typically be obtained during magnetic resonance (MR) examination.

The following are advanced cardiovascular monitoring equipment:

1. *Electrocardiograph*. The ECG monitors the electrical activity of the heart. With each beat the atria and ventricles depolarize and repolarise. The depolarisation and repolarisation are synchronised in each chamber, and thus, the action potentials from each fibre summate, producing a signal that is large enough to be measured at the surface of the body. The electrical signal is picked up by electrodes, amplified and displayed on a screen. The ECG is always measured as the difference in voltage between two electrodes. Depending upon the placement of the electrodes, the ECG has different shapes. If the electrodes are placed on each arm, a lead I waveform is obtained. Lead II is measured from the right arm to the left leg, and lead III is measured from the left arm to the left leg. For anaesthetic monitoring, the lead configuration is largely irrelevant because detailed measurements of the complex heights are not generally needed, requiring a waveform that contains all the main components. A lead II type trace, with positive *P*, *R* and *T* waves, is usually chosen. In theory, only two electrodes are needed to record an ECG since it is the voltage difference between the two electrodes. In practice, a third ground electrode is needed to reject interference. Different ECG electrode systems used in imaging modalities are shown in Figure 4.

The electrocardiogram only monitors the electrical activity of the heart, and the heart rate is derived from this. They do not provide information about the mechanical function of the heart or the state of the circulation. However, they are important for the diagnosis and treatment of arrhythmias. They are useful in critical situations where the blood pressure has fallen so low that peripheral pulses are not palpable. During imaging, it is crucial to get a good signal for the gating systems to work efficiently. Motion artefacts during MR imaging can be eliminated or greatly reduced by employing gating systems during acquisitions of the MR data. By synchronising MR data collection with the electrocardiogram, images can be obtained at specific times during the animal’s cardiac cycle.

2. *Pulse oximeter*. The pulse oximeter measures arterial oxygenation. Pulse oximeters are very useful as they detect arterial hypoxemia that may be related to problems that occur in anaesthesia such as hypoventilation, airway obstruction and equipment-related problems. Hypoxia is a common and serious problem, and a pulse oximeter will detect hypoxemia long before the animal becomes cyanosed. The pulse oximeter consists of a probe, a light-emitting diode and a photo-detector, which can be applied above a superficial vessel such that the light is transmitted across the tissues. The emitted light alternately transmits red light at two different wavelengths (in the visible and infrared regions) which are absorbed to different degrees by oxyhaemoglobin and deoxyhaemoglobin. The intensity of transmitted light reaching the photo-detector is converted to an electrical signal. This information is processed, and the absorption due to the tissues and venous blood, which is static, is subtracted from the best-to-beat variation to display the peripheral oxygen saturation both as a wave form and a digital reading. The waveform can also be interpreted to give a reading of heart rate. Strictly speaking, the pulse oximeter relies on and therefore gives information about both circulatory and respiratory systems.

**Figure 5:**
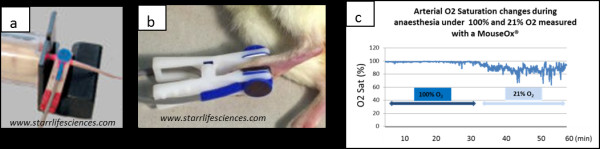
** Images displaying the clip sensors used by the pulse oximeter systems.** (**a**) In the base of the mouse or (**b**) in the centre of the foot in rat. The MouseOx® murine pulse oximeter system from Starr Life Sciences® Corp. (Oakmont, PA, USA) provides measurements of O_2_ saturation, pulse rate, respiration and pulse and breathe distension. (**c**) Profile of arterial O_2_ saturation measurement in rat during MRI acquisitions at 100% and 21% O_2_ during inhalation anaesthesia with isoflurane.

For laboratory animals, the light sensor clips have been adapted to be placed onto the animal’s thigh, foot, neck (mostly for mice) or tail for rats and larger rodents (Figure 5), and assesses the colour of arterial blood close to the surface. The system provides real-time continuous measurements of arterial O_2_, saturation, pulse strength, breath rate, blood flow and effort to breathe.

The absolute accuracy of pulse oximeters seems to be a little lower for animals than for humans [56], but the trends are still important, and overall, this equipment is a very valuable tool for monitoring animals. In general, a saturation of >95% is good. If it falls to <90%, then the anaesthetist should take note, but corrective action may not be necessary, especially if the cause is known and self-limiting. When the saturation falls below <80%, action should be taken to improve oxygenation. Some pulse oximeter systems are also MRI compatible with adapted non-ferrous-magnetic clips and cables for monitoring animals while imaged in small bore preclinical MRI scanners [57] (Figure 5).

3. *Blood pressure*. The blood pressure measurement is one of the best methods to monitor the cardiovascular system during anaesthesia [58]. The mean arterial pressure (MAP) is the best indicator of how well tissues are perfused. As the MAP falls, vital organ auto-regulation and perfusion is lost. The susceptibility of tissues to poor circulation varies tremendously depending upon their metabolic rate. The MAP in anaesthetised mice drops is around 80 ± 10 mmHg [59], with average systolic pressures of 112 ± 10 and mean diastolic pressures of 42 ± 12 mmHg. Combinations of injectable anaesthetics such as ketamine and xylazine induce marked decrease of the MAP and the pulse rate [37]. Similarly, isoflurane also has coronary vasodilatation properties and thus can alter blood flow parameters in rodents [60]. Therefore, blood pressure monitoring remains a very important process especially when undertaking functional MRI studies [61] or when assessing the perfusion of radiotracers through specific tissue/organs during PET/SPECT imaging.

**Figure 6:**
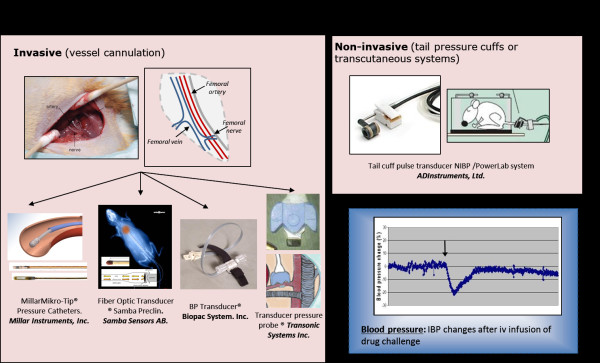
** Several systems available for monitoring blood pressure.** They include Millar probes (Millar Mikro-Tip®, Millar Sensors Systems, ADInstruments Ltd., Oxford, UK), fibre optic transducer, (which are most suitable for MRI imaging (e.g. Samba Preclin catheter®; Samba Sensors AB, Västra Frölunda, Sweden) and pressure transducers (TSD104A blood pressure transducer; Biopac Systems, Inc., Goleta, CA, USA). The perivascular flow probes (Transonic Systems, Inc., Ithaca, NY, USA) provide good precision blood pressure measurements for small animal vessels (0.25 mm) without the need to expose the lumen of the vessels, although the procedure still requires surgical exposure of the vessels to localise and fix the transducer around the vessel wall [64]. Indirect blood pressure method (non-invasive) involves inflating a cuff around the tail which works as a pressure transducer measuring the blood flow in the tail artery.

There are two basic methods for measuring arterial blood pressure, direct and indirect. The direct blood pressure measurement involves placing a catheter in an artery and connecting it to a transducer. In rodents, this procedure is invasive as the artery, femoral or carotid generally has to be exposed surgically. Once the catheter has been placed in the vessel and secured, it is either connected to a pressure transducer via a fluid-filled line or can be directly connected through the use of indwelling catheters and pressure sensing transducers [62]. For the fluid filled systems, specific small lines are used for rodents to minimise mobilising large volumes of blood/fluid to minimise any site effects on the animal’s homeostasis [26] An alternative system can be used for blood pressure in conscious mice. It consists of a pulse transducer and inflatable tail pressure cuff configured for use with rodents. The system records both the cuff pressure and pulse transducer signal from the tail artery [63]. Several systems available are shown in Figure 6.

#### Body temperature

Most anaesthetic agents profoundly depress thermoregulation, and laboratory rodents with a high metabolic rate and large surface area-to-body weight ratio are likely to suffer hypothermia. This is particularly critical when anaesthetising mice and can be a source of considerable mortality; therefore, core temperature monitoring is highly recommended. Temperature can be monitored using a digital rectal thermometer or thermocouple; there are several systems available in the market for all the imaging modalities. Due to muscle relaxation, rectal temperature may be up to 1°C less than the core body temperature. Hypothermia can be treated using an external heat source, with several systems available including circulating hot water blankets, blowing air systems, electric pads, bubble wrap or infrared lamps. Hyperthermia is as dangerous as hypothermia; thus, the external heat source should not only be ‘thermostatically controlled’ but should be linked to the core temperature. It is important to minimise heat losses during the animal preparation before imaging (e.g. hair removal, alcohol-based surgical preparation) using regulated heating pad and/or by covering the animal, in particular, the tail, and insulating it from a cold surface. If fluid replacement is required, the isotonic solutions should be warmed to 37*°*C.

#### Colour mucous membrane, capillary refill time and assessment of the central nervous system awareness

These parameters are generally challenging to assess during imaging while the animals are placed within the scanners, which are not accessible to researchers. During induction of anaesthesia, most animals will become ataxic, lose their righting reflex and eventually become immobile. At this depth of anaesthesia, they can easily be roused by painful stimuli, so anaesthesia must be allowed to deepen until such responses to pain are absent. To assess the depth of anaesthesia, the researcher can check the muscle tone and the response to reflexes of the anaesthetized animal. Some other regularly checked reflexes include the foot pinch stimulus (loss of the pedal withdrawal response) and assessing the blink response (palpebral reflex) of the animals.

### Long-term anaesthesia and monitoring recovery

Some imaging protocols, in particular for MR imaging, may require long acquisition times, and thus, the animal will have to be under anaesthesia for a long time. When animals are anaesthetised for a long period of time, adverse effects caused by poor technique and/or side effects of many anaesthetic drugs become more apparent and increasingly critical for the animal’s well-being and the experimental outcome. Therefore, it is very important that all the monitoring procedures abovementioned are well implemented and thoroughly applied during long-term imaging.

Anaesthesia can be prolonged by maintaining the animal on inhalation anaesthesia or by repeating standard injectable regimes. Metabolites will accumulate in an escalating manner, so it is important to assess how this can interfere with the purpose of the experiment.

To minimise systemic accumulation of anaesthetic metabolites and detrimental effects of long-term use of anaesthetic drugs, three regimes are generally used for long-term anaesthesia:

1) Using minimally metabolised volatile anaesthetic gases (e.g. isoflurane). There is very little practical difference between administering an inhalation agent for 30 min or for 8 h. Once the animal has been anaesthetised, it will remain anaesthetised if supplied with an appropriate maintenance concentration of anaesthetic. After 1 or 2 h, the maintenance concentration of anaesthetic can usually be reduced; further reductions may be possible, particularly if the animal is stable, and no further painful stimulus is given. The major problems encountered when using volatile anaesthetics for prolonged periods is usually due to anaesthetic techniques or equipment failure. It is essential to select an anaesthetic circuit with minimal dead space and minimal circuit resistance: this is particularly critical for the MR systems in which the gas circuit is generally quite long to access the animal in the magnet bore.

2) Continuous intravenous infusion with rapidly metabolised and eliminated agents such as propofol. There are many problems associated with prolonging anaesthesia using repeated doses of an injectable agent at frequent time intervals. Giving intermittent doses of the drug will cause the depth of anaesthesia to vary considerably, although this may be overcome by administering it as a continuous infusion so that steady plasma concentrations of the anaesthetic are maintained. However, this will require good monitoring of the animals, familiarisation with the anaesthetic regime and the pharmacodynamics of the anaesthetic agent. Animals will have to be catheterised, and a long infusion line may be required to reach the animal while placed inside the scanner during imaging (e.g. for MRI). In general, repeated doses of anaesthetic drug will increase the depth and duration of the anaesthesia state, which could potentially prolong the recovery time. It is preferable to administer incremental doses of the drug by intravenous route as the effects are seen more rapidly, and also, the dosing/effect can be more accurately adjusted. Administration by other routes is possible, but the depth of anaesthesia will vary less predictably.

3) Long-acting injectable anaesthetic agents. An alternative to administering repeated doses or a continuous infusion of short-acting agents is to select an anaesthetic with a prolonged duration of action (e.g. urethane, *α*-chloralose).

It is also critical to maintain normal physiology during long-term anaesthesia. In addition to the general monitoring guidelines already mentioned, there are some important procedures that should be followed to ensure the homeostasis of the long-term anaesthetised animal:

1) *Regular repositioning*. To avoid any damage that can be caused to muscles and nerves by the long-term immobilised position and/or the adoption of an abnormal position during long-term anaesthesia, it is prudent to reposition the animal regularly (every few hours) to minimise local pressure effects and aid general circulation. Unfortunately, this may not always be feasible in some imaging protocols in which the animal is required to maintain the same position for the acquisitions. In this case, it is important to ensure that animals are placed in a proper imaging bed. The animal can be immobilised, but excessive pressure should not be placed on it, and normal physiological movements (e.g. breathing) should not be compromised.

2) *Eye protection*. The eyes should be protected both from exposure keratitis (by regular application of ophthalmic ointment e.g. Lacrilube) and from retinal damaged caused by exposure to excessive light intensity in albino strains.

3) *Temperature*. Core temperature monitoring and maintenance is essential during long-term anaesthesia; hypo- or hyperthermia will interfere with many electrophysiological outcome measurements. The extremities and tail should be covered whenever possible.

4) *Blood sampling*. Blood sampling for blood gases, electrolytes, pH, haemoconcentration etc. is ideal for long-term monitoring, and correction for any abnormality by means of the appropriate electrolyte administration, but this is not always feasible in small rodents. However, end tidal capnography should be used, and the ventilator adjusted to keep end expired CO_2_ as near normal as possible. If the animal is kept at normal hydration and temperature, its kidneys should maintain normal homeostasis. In the extreme situation of extensive long-term anaesthesia (after 24 h), the risk for oxygen toxicity (muscle fasciculation, convulsion) is increased, and in this case, the inspired oxygen should be kept below 40%.

5) *Fluid status and urine output*. Physiological fluids such as Hartmann’s (e.g. lactated Ringer’s alternating with glucose-saline) should be infused in order to meet the daily fluid requirement and to maintain urine output at about 1to 2 ml/kg/h and to allow for respiratory losses. Unfortunately, this may be quite challenging due to the position of the animal in the imaging bore and experimentally required blood/fluid collection.

6) *Ventilation*. Some studies will require the animal to be mechanically ventilated. Small animal ventilators are designed to achieve controlled ventilation of the animal’s lungs by the application of intermittent positive pressure to the airway, providing the tidal volume and physiologically relevant respiratory rates. During mechanical ventilation, the ventilator applies a positive pressure to the anaesthetic gases to overcome airway resistance and elastic recoil of the chest, and flow occurs into the lungs (as compared to the negative intrathoracic pressure that draws air in during spontaneous ventilation). The technique is usually referred to as intermittent positive pressure ventilation. In both, spontaneous and mechanical ventilation expiration occurs by passive recoil of the lungs and chest wall.

When using the ventilator, it is important to set the right parameters for the animal species and size, accounting for the following: the breathing rate, the maximum inspiratory pressure and/or volume and the inspiratory-to-expiratory ratio (usually set at a 1:1 or 1:2). The latter can be increased to 1:3 or 1:4 to induce less cardiac depression, while maintaining inflation pressure below 15 cm H_2_O. This is because cardiac output is decreased when the thoracic pressure is positive (i.e. during inspiration when mechanically ventilated).

It is important to understand that mechanical ventilation reverses natural breathing, with important effects on thoracic haemodynamics and also overrides autonomic reflex control of breathing, which normally maintains blood gas homeostasis. When monitoring a ventilated animal, it is important to realise that carbon dioxide is the main stimulus for respiration and that, if the arterial carbon dioxide level is reduced below 30 to 35 mmHg, the animal will not attempt to breathe. A common mistake is to under-ventilate so that the animal fights the ventilator; this may be misinterpreted as the animal being not deep enough under anaesthesia and more anaesthetic is given. The end result is a hypercapnic animal that is too deeply anaesthetised. Ideally, one should have oxygen/carbon dioxide monitoring equipment (e.g. pulse oximeter, end tidal capnograph). The ventilation settings can then be adjusted to try to maintain physiological levels of O_2_/CO_2_. Blood gas and electrolyte analysis are useful but confer intermittent monitoring, and a local analyser is needed. If the animal’s kidneys are functioning normally, (i.e. not shut down due to dehydration, blood or heat loss), the animal can maintain its blood electrolyte, and acid and base balance, provided that the ventilation rate is adjusted to maintain the CO_2_ level. For long-term protocols, this may involve fluid infusion. For long-term mechanical ventilation, it may be worth considering humidifying the inspired gas mix to minimise airway drying and irritation. Regular tracheal aspiration may be needed to remove respiratory tract exudates from the endotracheal tube in larger laboratory animal species, or alternatively, an anti-muscarinic agent such as atropine could be incorporated as a premedication.

#### Recovery

Monitoring should be carried out until the animal has fully recovered and there is confidence that all essential physiological functions are well recovered, in particular, normal respiratory and cardiovascular function, body temperature and general behaviour. It is very important to carry out such assessments before the animal is transferred back to its housing cage.

· *Assessment of peripheral perfusion.* Assessment of peripheral perfusion through the colour mucous membrane and capillary refill time since perfusion decreases with anaesthesia. If the colour of the mucous membranes is pale or cyanotic, it is important to check the cardiovascular and respiratory systems as abovementioned. Excessive loss of fluids through bleeding or evaporation if a body cavity is surgically opened will rapidly lead to complications such as circulatory shock. This in turn will affect the function of many organs in a manner that could have an impact on research data. If substantial fluid loss is anticipated, calculation of fluid replacement requirements can be estimated by weighing the animal before and after the procedure. Lost fluids can be replaced using solutions such as 0.9% saline or lactated Ringer’s solution, preferably warmed to 37°C and administered subcutaneously or intraperitoneally.

· *Assessment of central nervous system awareness.* This is important for assessing the depth of the anaesthesia and, in particular, the level of consciousness and immobilisation/responsiveness of the anaesthetised animal during recovery. After administration of some anaesthetic agents, especially injectable agents, animals may become ataxic, show poor coordination and poor reflex response, especially on the back limbs during the recovery, and therefore, it is important that they are monitored closely until they gain normal behaviour.

During recovery, it is important to maintain the core temperature of the animal, placing the animals on their right side or in sternal recumbency, in a warm recovery cage with no sawdust or suchlike bedding that may be inhaled. If the animals have been under anaesthesia for a relatively long time, it may be worth considering to supply oxygen and fluids and nutrition supplement (oral or parenteral support e.g. high-energy moist foods such as nutrient agar, jelly or crushed rodent pellets mixed with water) presented in a petri dish or other manner that does not require the animal to reach up high. Additional analgesia should be provided if the animal has undergone a painful procedure or if there appears to be any signs of pain. It is very important to assess for any signs of pain, e.g. guarding (e.g. limping) or licking/rubbing the affected area, quietness, isolation from the group, failure to explore, hunched posture, piloerection and weight loss [64].

## Conclusions

Rodent imaging tools permit new investigations to be made into fundamental biology, and answers to these queries often reveal a need for more imaging tools with which to pursue additional new knowledge, that is, new tools and new findings reinforce each other, leading to an ever-advancing frontier of new knowledge about human health and disease obtained through imaging research with rodents. Laboratory rodent imaging is a relatively new field which is expanding very rapidly and being integrated into a wide variety of biomedical disciplines. Therefore, it is important to consolidate sound protocols for handling, anaesthesia and monitoring the animals to ensure suitability for all the specific needs of a wide range of imaging experiments. Obtaining accurate and consistent images remains one of the major challenges in preclinical imaging, in particular, for longitudinal studies. Success relies extensively on choosing the appropriate anaesthetic regime and using suitable monitoring equipment to assist in maintaining good homeostasis. While anaesthetic monitoring equipment for rodent imaging is in its infancy, it is anticipated that this rapidly advancing field holds great opportunities for further research and technology development. Indeed, further refinement of animal handling protocols, implementation of new anaesthetic regimes and development in monitoring equipment are needed to maximise the potential of current preclinical imaging systems, minimising any confounding effects on the imaging outcomes and the studied biological processes. It also represents an important step towards improving animal welfare and the three Rs’ principles of humane experimental technique: replacement, refinement and reduction.

## Competing interests

The authors declare that they have no competing interests.

## Authors’ contributions

JLT performed some of the reported animal experiments, reviewed current literature, provided the concept for the review and wrote the manuscript. AK provided very valuable intellectual advice on rodent anaesthesia and critically edited and reviewed the manuscript. WG reviewed the manuscript and supported the conception of this review. All the authors read and approved the final manuscript.
